# Assessment of the Degree of Erythema Reduction in Rosacea After Polychromatic Light Treatments

**DOI:** 10.3390/jcm15010302

**Published:** 2025-12-31

**Authors:** Anna Deda, Aleksandra Lipka-Trawińska, Dominika Wcisło-Dziadecka, Bartosz Miziołek, Magdalena Hartman-Petrycka, Sławomir Wilczyński

**Affiliations:** 1 Department of Practical Cosmetology and Skin Diagnostics, Faculty of Pharmaceutical Sciences in Sosnowiec, Medical University of Silesia in Katowice, 41-200 Sosnowiec, Poland; 2 Clinical Department of Dermatology, Department of Internal Medicine, Dermatology and Allergology, Faculty of Medical Sciences in Zabrze, Medical University of Silesia in Katowice, 41-800 Zabrze, Poland; 3 Clinical Department of Dermatology, Faculty of Medical Sciences in Katowice, Medical University of Silesia in Katowice, 40-752 Katowice, Poland; 4 Department of Basic Biomedical Science, Faculty of Pharmaceutical Sciences in Sosnowiec, Medical University of Silesia in Katowice, 41-200 Sosnowiec, Polandswilczynski@sum.edu.pl (S.W.)

**Keywords:** rosacea, Intense Pulsed Light (IPL), erythema reduction, Grey Level Co-Occurrence Matrix (GLCM), Clinician Erythema Assessment (CEA)

## Abstract

**Background:** Rosacea is a chronic facial skin disease in which persistent erythema is a significant clinical problem, often resistant to standard therapies. Intensive pulsating light (IPL) has become a recognised and effective method of treating erythema and telangiectasia. The latest recommendations emphasise the advantage of combining subjective clinical assessments with objective imaging analyses in monitoring therapy effects. **Methods:** A total of 20 patients with rosacea qualified for this study. They were subjected to three polychromatic light procedures (Lumecca, Inmode; wavelength of 515–1200 nm) at 21-day intervals. The skin condition was documented photographically, and the degree of erythema was assessed on the basis of the Clinician Erythema Assessment (CEA) scale and objective analysis of the skin texture, using the parameters of contrast and homogeneity of the grey level co-occurrence matrix (GLCM). **Results:** A series of three polychromatic light treatments yielded a significant clinical improvement in all patients. The mean CEA value decreased by 61.11%, whereas the GLCM contrast in all the analysed facial areas dropped by about 17%, and homogeneity increased by 4–5%. The effects persisted for at least three months after the treatments. A high correlation of CEA scale results with GLCM parameters (R = 0.81–0.94 for contrast; R = −0.77 to −0.83 for homogeneity) was observed. **Conclusions:** Three polychromatic light treatments proved to be a very effective method of reducing erythema in rosacea, confirmed by both clinical evaluation and objective imaging analysis. The effects of therapy were durable and clear. Integration of the subjective method (CEA) with GLCM analysis can be a path for future research and clinical practice in the assessment of erythematous skin lesions.

## 1. Introduction

Rosacea is a chronic, recurrent, inflammatory skin disease, mainly affecting central parts of the face. It is characterised by recurrent or fixed erythema, telangiectasias, papulae, pustulae and hypertrophic changes. Secondary symptoms such as itching, burning or stinging are often observed in patients with rosacea. Erythema, both temporary (with redness) and fixed, is a clinical feature of the most common subtype, namely erythematotelangiectatic rosacea. It is also characteristic of a papulopustular variant and can accompany hypertrophic changes. Rosacea, due to its location on the face, is often the source of a significant psychosocial burden for people affected by this disease [1,2,3,4,5,6].

The pathophysiology of erythema in rosacea is multifactorial and has been only partially explained. A key role is played by vascular dysregulation, including recurrent vascular dilatation and increased blood flow to surface layers of the skin. This vascular instability is often caused by environmental stimuli (such as temperature changes, exposure to UV radiation or spicy dishes), but it also results from innate dysregulation of the neurovascular and immune systems [2,3,7,8,9]. The latest research indicates the participation of both congenital and adaptive immune networks, with irregularities in receptors recognising patterns, such as Toll-like receptor 2 (TLR2), increased expression of epidermal proteases, increased levels of pro-inflammatory cathelicidin peptides and increased cytokine/chemokine expression, which ultimately promotes the expansion of blood vessels and chronic inflammation [10,11]. Factors such as disturbed skin microbiota and density of Demodex folliculorum mites can additionally exacerbate the inflammatory cascade and vascular lesions, thus intensifying erythema. Over time, these processes can cause permanent dilatation of skin vessels and, in advanced cases, skin fibrosis [11,12,13].

Persistent face erythema often becomes resistant to conventional local and systemic therapies, which has prompted the search for energy-based methods. Intensive pulsating light (IPL) has turned out to be an effective and well-tested option for treating rosacea-related erythema [14,15,16,17]. The mechanism of action consists of selective photothermolism of oxyhaemoglobin in surface blood vessels, which leads to their destruction and a significant reduction in visible erythema and telangiectasia. Unlike lasers, IPL emits non-coherent light of high intensity, with wavelengths from 500 to 1200 nm. Depending on the filters used, various light spectra that allow for selective tissue damage are obtained. For example, longer waves aim at deeper vessels, whereas shorter ones aim at surface vessels. In addition, combinations of one specific wavelength with fluency, duration of the impulse and impulse spacing ensure the versatility of the device and make it possible to affect many chromophores and skin structures, which translates into a wide range of treatments for skin disorders, such as pigmentation changes, photoaging and unwanted hair [18,19]. Clinical studies consistently show a significant improvement in erythema intensity and satisfaction of patients after a series of IPL treatments. The effects usually persist for at least a few months, and the risk of side effects is minimal. Effectiveness can be even higher in patients with papulopustular subtypes, although benefits can be observed in all cases [16,17,18].

The assessment of erythema reduction in clinical trials is based on both subjective and objective tools. The most commonly used tools include the Clinician Erythema Assessment (CEA). It is a standardised, five-degree scale widely used in both clinical trials and in everyday practice, enabling semi-quantitative, repetitive assessment of the degree of facial erythema based on a medical examination [20,21,22]. However, clinical observation can be subjective by nature and susceptible to inter-observer variability. To overcome these limitations and increase objectivity, modern research is increasingly using instrumental and digital image analysis techniques. Among these, the grey level co-occurrence matrix (GLCM) has gained popularity as a solid method of quantitative analysis of skin texture and erythema [23,24,25,26]. GLCM analysis measures spatial relationships and patterns of pixel intensity distribution in standardised photographs, calculating parameters such as contrast and homogeneity. Higher contrast and lower homogeneity in GLCM analyses are associated with increased irregularity and intensity of pigmentation and/or erythema, whereas effective treatment should result in reduced contrast and increased homogeneity—objective clinical improvement indicators [23,24,25,26,27,28,29,30]. Integration of advanced imaging analyses, such as GLCM, is a valuable complement to traditional clinical scales, increasing the precision of the assessment of results in both scientific research and medical practice.

To sum up, persistent erythema is a complex, multifactorial symptom of rosacea, closely related to vascular and immune disorders. Accurate assessment and monitoring require both subjective assessment and objective, quantitative methods. IPL phototherapy is a proven and effective method of reducing erythema, and the integration of advanced imaging analyses, such as GLCM, is a valuable complement to traditional clinical scales, increasing the precision of the assessment of results in both scientific research and medical practice.

The aim of this study was to assess the effectiveness of reducing erythema using polychromatic light in patients affected by rosacea. Another aim was an attempt at assessing the correlation between the subjective Clinician Erythema Assessment scale, commonly used for evaluating the efficiency of rosacea therapy, and imaging analyses, such as GLCM.

## 2. Materials and Methods

### 2.1. Study Participants

A total of 20 participants (15 women and 5 men) aged 23 to 68 years were included in this study. The mean age was 40.70 ± 12.84 years. The patients suffered from erythema telangiectasia rosacea. In 5 of them, features of a papulopustular variant were also observed. One patient had hypertrophic changes within the nose. All patients had previously used local rosacea therapy, some patients (11 subjects) had previously taken oral tetracyclines, and 2 patients had taken oral isotretinoin.

The exclusion criteria included patient’s age below 18 years of age; susceptibility to keloids and hypertrophy of scars; pregnancy and breastfeeding; use of hormone therapy; use of steroid drugs up to three months before treatment; use of vitamin A derivatives topically up to one month before treatment or orally up to six months before treatment; use of local and/or oral antibiotics up to three months before treatment; performance of chemical peels up to one month before treatment; use of laser treatments up to three months before treatment; inflammatory skin conditions other than rosacea; autoimmune conditions; viral, bacterial and fungal diseases of the skin; use of drugs or herbs with photosensitising properties; and surgical procedures in the treatment area in less than 3 months. Before starting polychromatic light therapy, a dermatologist conducted a medical interview and qualified the volunteers for treatments.

This study was approved by the Bioethics Committee (Medical University of Silesia No. PCN/0022/KB1/11/I/20 of 19 May 2020). This study was conducted in accordance with the principles of the Declaration of Helsinki.

### 2.2. Procedure of Erythema Reduction Treatment

Each volunteer was subjected to three treatments aimed at reducing erythema and telangiectasia lesions using Lumecca, Inmode (Irvine, CA, USA). An applicator emitting light with wavelengths in the range of 515–1200 nm was used; the impulse duration was dependent on the energy density and amounted to its 1/3 in milliseconds. The manufacturer declares that 40% of emitted radiation is absorbed by haemoglobin. The parameters of the procedure were selected individually depending on the skin reaction. In the first treatment, the mean energy density was 12.7 ± 0.8 J/cm^2^; in the second treatment, the mean energy density was 14.5 ± 0.76 J/cm^2^; and in the third one, it was 16.1 ± 0.64 J/cm^2^ (detailed information can be found in Table 1). The applicator was run across the skin only once each time. At the end of the treatment, a sunscreen with SPF 50+ was applied. The volunteers were also informed about the need to use sunscreens. The treatments were performed with an interval of 21 days.

### 2.3. Photographic Documentation

Each volunteer had skin images acquired using the Fotomedicus system (Elfo, Łódź, Poland) before the first treatment, 4 weeks after 3 treatments and 3 months after 3 treatments. Study participants had a series of clinical photographs taken in non-polarized light for documentation purposes and in cross-polarized light for further image processing and analysis. Cross-polarization of light makes it possible to reduce the scattering and reflection of light from the sebaceous layer on the skin as well as allows for better visualisation of changes in the epidermis and skin, which are important for the analysis of the severity of erythema and vascular lesions.

The photographic documentation obtained using the Fotomedicus system was subjected to GLCM (Grey Level Co-Occurrence Matrix) analysis.

### 2.4. GLCM Analysis

GLCM (Grey Level Co-Occurrence Matrix) analysis identifies how often a pixel with a certain brightness is situated next to a pixel of a differing brightness in an image. Erythematous lesions become most prominent when the contrast between the affected area and the surrounding healthy skin reaches its maximum. As a consequence, the way these erythematous regions are perceived subjectively depends on factors such as the number of erythema spots in a given area, the intensity of their colouration and the contrast between the lesion and healthy skin [31,32].

Within the GLCM matrix, both rows and columns represent the number of distinct grey levels (G) in the image. Image evaluation may be conducted across various directions—vertical (90°), horizontal (0°), or diagonal (45° or 135°) [32,33]. In this analysis, pixels positioned horizontally (θ = 0°) and immediately adjacent (distance d = 1) were assessed.

The performed GLCM analysis included contrast and homogeneity.

**Figure 1:**
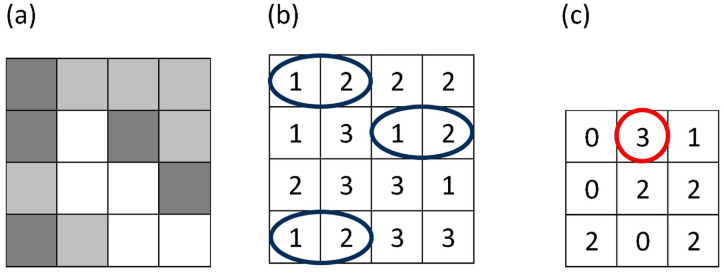
Figure illustrates how a gray-level co-occurrence matrix (GLCM) is constructed from a small grayscale image (**a**). In panel (**b**), the blue ellipses indicate all horizontally adjacent pixel pairs with gray levels (1,2). In panel (**c**), the red circle marks the GLCM element for the pair (1,2), whose value (3) shows that this pair appears three times in the image for the chosen offset.

The GLCM analysis focused on measuring contrast and homogeneity. According to the defined formula, GLCM contrast quantifies the variation in brightness among neighbouring pixels, offering an evaluation of how frequently pixels of specific brightness levels appear next to each other. As an illustration, Figure 1 demonstrates the frequency of occurrence—in the horizontal direction—of pixels with brightness levels 1 and 2 being adjacent. For the images studied, GLCM had a dimension of 256 by 256, reflecting the use of 8-bit greyscale depth [32,33]. For each analysed region of interest, a grey level co-occurrence matrix was constructed, in which *P*(*i*,*j*)*P*(*i*,*j*)*P*(*i*,*j*) denotes the normalised probability of the co-occurrence of grey levels *iii* and *jjj* at a given spatial relationship in the image.

$$\sum_{i,j}P_{i,j}{\left(i-j\right)}^{2}$$

where

*i*—brightness of the tested pixel;

*j*—brightness of the adjacent pixel.

All cross-polarized images were converted to 8-bit greyscale (256 grey levels) and four ROIs (the spots including erythematous lesions): left cheek, right cheek, nose and chin were manually delineated for each subject. For each ROI, a GLCM of dimension 256 × 256 was generated using a horizontal direction of 0° with a pixel distance d = 1.

The analysis of the GLCM homogeneity of the image was aimed at determining the homogeneity of pixel brightness within the entire ROI—i.e., the intensity of lesions in the entire studied spot. Homogeneity in the adopted research model was understood as

$$\sum_{i,j}\frac{P(i,j)}{1+\left|i-j\right|}$$

where

*i*—brightness of the tested pixel;

*j*—brightness of the adjacent pixel.

Homogeneity for the whole face was derived as a weighted combination of ROI values (0.35 for each cheek and 0.15 for the nose and chin). The greater the homogeneity and the smaller the contrast, the greater the homogeneity of the skin, i.e., the intensification of erythematous and vascular lesions decreases. Contrast and homogeneity are given in relative units (as above).

Four ROIs were delineated in the GLCM image analysis: left cheek, right cheek, nose and chin. The impact of the treatments on the individual facial zones was analysed using the values obtained in each ROI. The analysis of the whole face, on the other hand, was performed using the values calculated from the following formula: GLCM contrast of the entire face = GLCM contrast of the left cheek × 0.35 + GLCM contrast of the right cheek × 0.35 + GLCM contrast of the nose × 0.15 + GLCM contrast of the chin × 0.15, and homogeneity of the entire face from the formula GLCM homogeneity of the entire face = GLCM homogeneity of the left cheek × 0.35 + GLCM homogeneity of the right cheek × 0.35 + GLCM homogeneity of the nose × 0.15 + GLCM homogeneity of the chin × 0.15.

The GLCM algorithms operating in the MATLAB Version 7.11.0.584 (R2010b) environment were used for the quantitative analysis of vascular lesions.

### 2.5. Clinician Erythema Assessment (CEA)

The Clinician Erythema Assessment (CEA) is a semi-quantitative tool for assessing the severity of facial erythema, especially in patients with rosacea. It is a 5-degree scale from 0 to 4, where 0 means no visible erythema; 1 means very mild erythema, barely noticeable; 2 means mild erythema; 3 means moderate erythema; and 4 means strong, intense redness. The scale is based on a visual assessment by a trained clinician who assesses the intensity and extent of erythema in the central part of the face, often focusing on the cheeks, nose and chin. The CEA aims to ensure an objective and practical assessment of erythema for clinical and research purposes [20,22]. The assessment is carried out under standard conditions, with appropriate lighting to minimise variability. Erythema is usually assessed by trained dermatologists, which ensures consistency and repeatability of the results. The use of the CEA scale allows doctors to track changes in the severity of erythema over time, especially to assess treatment effectiveness. The CEA scale has been confirmed by tests showing high inter- and intra-observer reliability, which means that various doctors usually agree on the use of the scale and individual doctors provide consistent results in repeated assessments [20]. Such reliability emphasises its usefulness in clinical studies and routine practice in monitoring rosacea-related erythema.

### 2.6. Statistical Analysis

The statistical analysis was carried out using the Statistica 13 program. The following variables were included in the analysis: Clinician Erythema Assessment (CEA) scores evaluated by three dermatologists before the treatments, four weeks after three treatments and three months after three treatments in 20 subjects; GLCM contrast of 4 facial areas subjected to analysis before the treatments, four weeks after three treatments and three months after three treatments in 20 subjects; GLCM contrast of the entire face before the treatments, four weeks after three treatments and three months after three treatments in 20 subjects; GLCM homogeneity of 4 facial areas subjected to analysis before the treatments, four weeks after three treatments and three months after three treatments in 20 subjects; and GLCM homogeneity of the entire face before the treatments, four weeks after three treatments and three months after three treatments in 20 subjects. The lack of normality of distribution was shown in the Shapiro–Wilk test. The ANOVA Friedman test was used to analyse the impact of treatments on the skin condition, and post hoc pairwise comparisons between time points (T0 vs. T1, T0 vs. T2 and T1 vs. T2, where applicable) were performed using the Dunn’s test. The Spearman rank correlation was used to assess the interdependence between the CEA scale-based assessments of experts and the GLCM contrast and homogeneity of the face. The results were considered statistically significant when *p* < 0.05.

## 3. Results

### 3.1. Treatment Effectiveness

**Figure 2:**
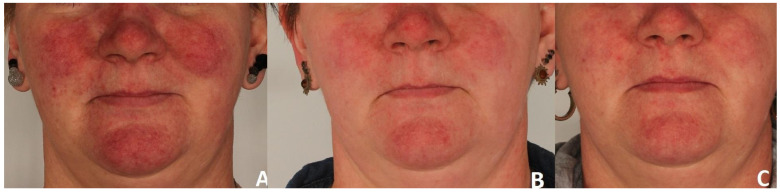
Erythema reduction in patient 1. Panel (**A**) represents the condition before treatment, panel (**B**) shows the skin four weeks after completion of three polychromatic light sessions, and panel (**C**) shows the skin three months after the three-session treatment series.

**Figure 3:**
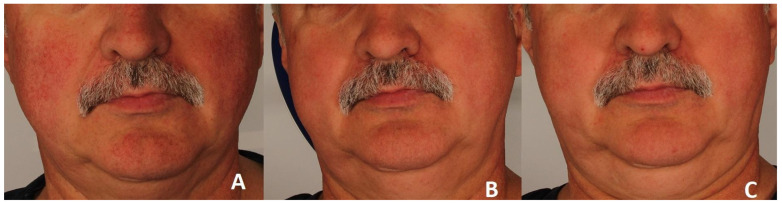
Erythema reduction in patient 2. Panel (**A**) represents the condition before treatment, panel (**B**) shows the skin four weeks after completion of three polychromatic light sessions, and panel (**C**) shows the skin three months after the three-session treatment series.

A significant clinical improvement in the condition of the volunteers’ skin was observed after a series of three polychromatic light treatments using the applicator emitting light in the range of 515–1200 nm (Figure 2 and Figure 3).

The assessment carried out by three independent experts using the CEA scale showed a considerable reduction in the intensity of erythematous lesions amounting to 61.11%. The subjective assessment of the doctors was confirmed by the results of the GLCM analysis.

**Figure 4:**
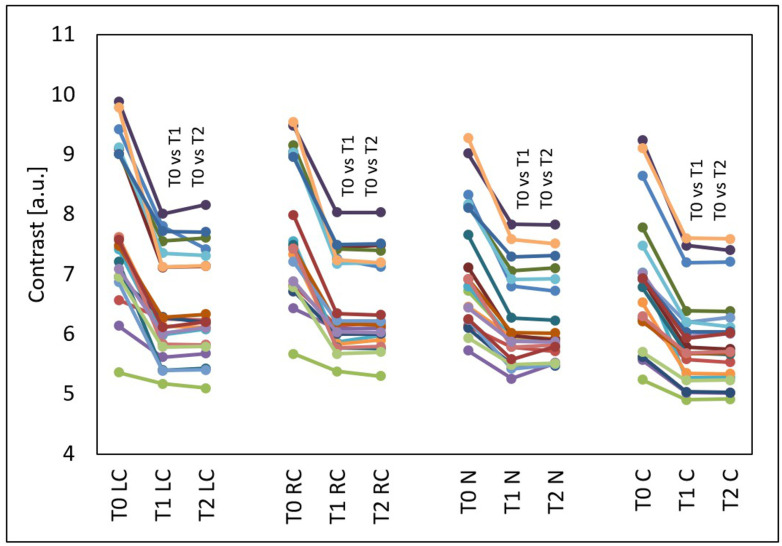
GLCM contrast of the left cheek (LC), right cheek (RC), nose (N) and chin (C) before the treatments (T0), four weeks after three treatments (T1) and three months after three treatments (T2), T0 vs. T1 and T0 vs. T2—statistically significant inter-group differences (post hoc, ANOVA Friedman).

The GLCM contrast decreased in all evaluated facial areas (*p* < 0.001) (Figure 4). This effect occurred in all test subjects and also persisted three months after the treatments. Compared to the time before the treatments (T0), at four weeks after three treatments (T1), the median GLCM contrast decreased by 17.61% from 7.52 to 6.20 on the left cheek (*p* < 0.05), by 17.44% from 7.46 to 6.16 on the right cheek (*p* < 0.05), by 13.35% from 6.86 to 5.95 on the nose (*p* < 0.05) and by 17.18% from 6.92 to 5.95 on the chin (*p* < 0.05). Compared to the time before the treatments (T0), at three months after three treatments (T2), the median GLCM contrast decreased by 17.60% from 7.52 to 6.20 on the left cheek (*p* < 0.05), by 17.29% from 7.46 to 6.17 on the right cheek (*p* < 0.05), by 13.70% from 6.86 to 5.92 on the nose (*p* < 0.05) and by 17.28% from 6.92 to 5.73 on the chin (*p* < 0.05).

**Figure 5:**
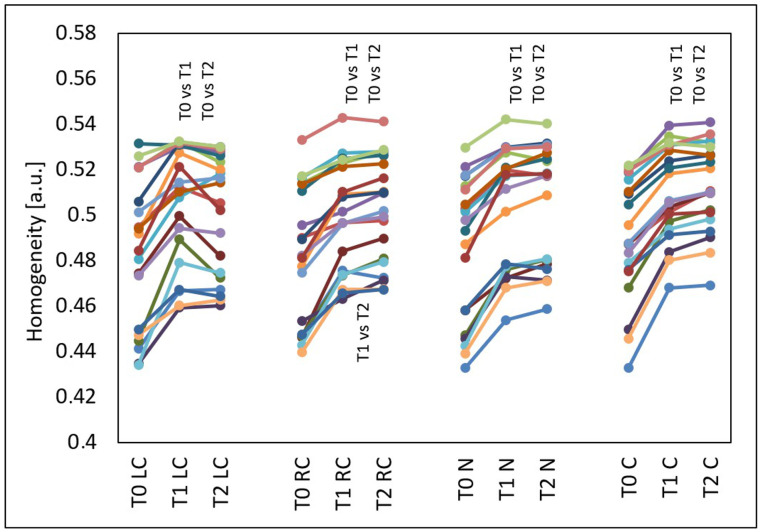
GLCM homogeneity of the left cheek (LC), right cheek (RC), nose (N) and chin (C) before the treatments (T0), four weeks after three treatments (T1) and three months after three treatments (T2), T0 vs. T1 and T0 vs. T2 and T1 vs. T2—statistically significant inter-group differences (post hoc, ANOVA Friedman).

The GLCM homogenicity increased in all evaluated facial areas (*p* < 0.001) (Figure 5). This effect occurred in almost all test subjects and also persisted three months after the treatments. Compared to the time before the treatments (T0), at four weeks after three treatments (T1), the median GLCM homogeneity increased by 4.71% from 0.488 to 0.511 on the left cheek (*p* < 0.05), by 3.61% from 0.482 to 0.499 on the right cheek (*p* < 0.05), by 4.45% from 0.496 to 0.518 on the nose (*p* < 0.05) and by 3.81% from 0.488 to 0.506 on the chin. Compared to the time before the treatments (T0), at three months after three treatments (T2), the median GLCM homogeneity increased by 4.42% from 0.488 to 0.510 on the left cheek (*p* < 0.05), by 4.99% from 0.482 to 0.506 on the right cheek (*p* < 0.05), by 4.50% from 0.496 to 0.518 on the nose (*p* < 0.05) and by 4.70% from 0.488 to 0.510 on the chin (*p* < 0.05). On the right cheek, there was also a difference in the GLCM homogeneity between the measurement time T1 (median 0.499) and T2 (median 0.506) (*p* < 0.05).

### 3.2. Comparison of the Results Calculated on the CAE Scale and Obtained from the GLCM Image Analysis

**Figure 6:**
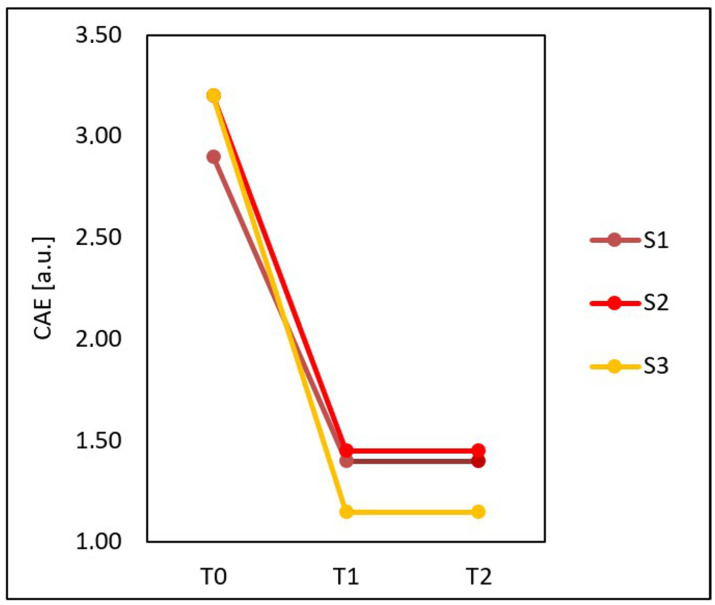
Mean CEA score calculated for the entire group examined before the treatments (T0), four weeks after three treatments (T1) and three months after three treatments (T2) obtained on the basis of assessments performed by three specialists: S1, S2 and S3.

Based on Figure 6, it can be observed that the mean CEA score calculated for the entire group examined before the treatments (T0), four weeks after three treatments (T1) and three months after three treatments (T2) was not fully compatible with the results of all the specialists. Specialist S1 at T0 granted some people lower CEA scores than specialist S3, and after the treatments, S3 awarded lower CEA scores than S1 and S2. The assessments of specialists S1 and S2 did not fully coincide before and after the treatments.

A total of 20 subjects participated in this study; each of them had the severity of erythema assessed three times, and 60 skin condition assessments were obtained. After calculating how many times the specialists at a given time assessed the skin condition identically, it turned out that they were in complete agreement in 39 assessments (65%), and in as much as 21 (35%), at least one specialist granted patients different CEA scores than the other two.

To compare the effects of the treatments evaluated by the specialists and obtained from the GLCM image analysis, the CEA scores of the specialists for each person were averaged, and the contrast for the entire face was calculated according to the following formula: GLCM contrast of the entire face = GLCM contrast of the left cheek × 0.35 + GLCM contrast of the right cheek × 0.35 + GLCM contrast of the nose × 0.15 + GLCM contrast of the chin × 0.15, and the homogeneity of the entire face according to the formula: GLCM homogeneity of the entire face = GLCM homogeneity of the left cheek × 0.35 + GLCM homogeneity of the right cheek × 0.35 + GLCM homogeneity of the nose × 0.15 + GLCM homogeneity of the chin × 0.15.

**Figure 7:**
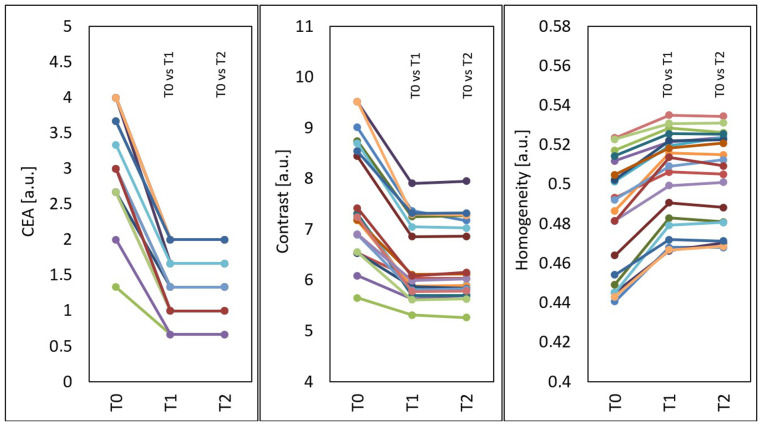
Mean CEA, GLCM contrast and GLCM homogeneity of the face before treatments (T0), four weeks after three treatments (T1) and three months after three treatments (T2), T0 vs. T1 and T0 vs. T2—statistically significant inter-group differences (post hoc, ANOVA Friedman).

Statistically significant changes were shown in all types of assessment of treatment effects. After the treatments, the mean CEA score (*p* < 0.001) and GLCM contrast of the face (*p* < 0.001) decreased, and the GLCM homogeneity of the face increased (*p* < 0.001) (Figure 7).

Compared to the time before treatments (T0), at four weeks after three treatments (T1), the median CAE decreased by 61.11% from 3.10 to 1.30 and remained the same at three months after three treatments (T2).

Compared to the time before treatments (T0), at four weeks after three treatments (T1), the median GLCM contrast of the face decreased by 17.33% from 7.28 to 6.02 (*p* < 0.05), and at three months after three treatments (T2), the median GLCM contrast of the face slightly increased by 0.22% compared to T1 and was 6.03 (*p* < 0.05) (decline by 17.11% in relation to the initial state).

Compared to the time before treatments (T0), at four weeks after three treatments (T1), the median GLCM homogeneity of the face increased by 4.49% from 0.490 to 0.512 (*p* < 0.05), and at three months after three treatments (T2), the median GLCM homogeneity of the face increased by 4.38% from 0.490 to 0.511 (*p* < 0.05).

The mean CEA score of the specialists statistically significantly correlated (*p* < 0.001) with the GLCM contrast and homogeneity of the face at T0, T1 and T2 (Table 2). Higher CEA scores translated into a greater GLCM contrast of the face, R = 0.94 T0, R = 0.84 T1 and R = 0.81 T2, and lower GLCM homogeneity of the face, R= −0.83 T0, R= −0.77 T1 and R= −0.79 T2.

Exploratory comparisons did not reveal any clear qualitative differences in treatment response between female and male participants in terms of CEA scores and GLCM parameters; however, these observations must be interpreted with caution given the small number of men.

## 4. Discussion

The results of this study showed the high effectiveness of a series of three polychromatic light treatments (wavelength of 515–1200 nm) in reducing erythema in patients with rosacea, which was confirmed by both the subjective Clinician Erythema Assessment (CEA) score and the objective analysis of skin texture using the parameters of GLCM contrast and homogeneity. A statistically significant reduction in the CEA score by over 60% was observed as well as a significant reduction in GLCM contrast and an increase in homogeneity, which persisted for at least three months after the treatments. These effects are confirmed by reports from the literature, according to which three IPL procedures were the optimal number of sessions ensuring a permanent improvement in erythema and vascular lesions, with minimal side effects and good patient tolerance. The study by Piccolo et al. [34] showed that three polychromatic light treatments at one-month intervals significantly improved the skin condition in more than 50% of volunteers with rosacea or other vascular lesions, and in 33% of patients, the improvement was good. Tsunoda et al. [35] obtained similar effects of improving the condition of the skin affected by vascular lesions. In their practice, they used two different IPL devices. First, a large laser spot (laser head sized 1 × 4 cm) emitting waves in the range of 590–1200 nm was run across the entire face, and then, a smaller laser spot (diameter of 6.35 mm) emitting waves in the range of 500–635 nm was run locally across areas affected by telangiectasia. In the assessment by 10 dermatologists, the mean improvement in the skin condition was 64.5%. The study by Kim et al. [15], comparing the effectiveness of short-impulse polychromatic light and dye laser in reducing the symptoms of rosacea after a series of three treatments, provided similar, very good therapy results. Dermatologists observed at least good improvement of >50% in 88.9% of patients.

The CEA scale is a commonly used and practical tool for assessing the severity of erythema in patients with rosacea. Clinical studies, such as the one by Tan et al. [20], confirm its high inter-observer reliability and good repeatability, especially when it is used by experienced dermatologists with previous training. However, our study revealed some discrepancies in the assessments of three specialists (65% of complete compliance), which indicates the natural subjectivity of this tool and its limitations resulting from a five-degree assessment scale, which does not allow for precise identification of minor clinical changes. For this reason, the use of averaged results of several experts and additional assessment methods is desirable to increase measurement objectivity and accuracy.

The GLCM analysis is a valuable, objective method of quantitative assessment of the skin texture features associated with erythema and inflammatory processes. Parameters such as contrast and homogeneity calculated on the basis of facial photos in polarized light measure spatial relationships between skin pixels, taking into account the irregularity and dispersion of erythematous colouring. Studies using optical imaging (OCT) and texture analysis in dermatology as well as RGB imaging (digital image reproduction techniques in which each pixel is defined by three components, namely red, green and blue) confirm that changes in these parameters correlate with the presence and severity of skin lesions, and their use can support diagnostics, monitoring of the course of diseases and assessment of therapy effectiveness [21,36,37]. In the literature, information can also be found on spectral analyses allowing for the assessment of erythema severity as well as digital analyses or fluorescent imaging [22].

The GLCM analysis is a tool that is increasingly used in medicine. There are reports about the possibility of using image analysis with this method to classify skin lesions (including skin cancer), detect tumours in radiological images, analyse images of blood and lungs or analyse cervical cytology. In dermatology, GLCM is used to classify skin cancer images and predict the spread of lesions based on texture [24,25,26,28,29,38] The study by Almeida et al. [25] confirmed the significant value of GLCM analysis when assessing medical images showing skin lesions. The research team obtained evidence that GLCM can effectively support differentiating between benign nevi and skin melanomas. The use of statistical methods based on GLCM features in combination with data from red, green and blue channels allowed for the analysis of tissue microtexture and classification of images in the context of cancer detection. The effectiveness of these methods was within 95–97%. In turn, Ansari et al. [28] used the GLCM method to extract key regions from dermatoscopic images of skin lesions, which were used to build a classifier recognising cancerous and healthy tissues. The developed system reached an accuracy of 95.00%. Pang et al. [29] proved that GLCM analysis is very useful when assessing changes in skin texture caused by the use of cosmetics and various types of cosmetic treatments—the texture was understood here as the spatial distribution and mutual relationships of pixel grey levels in the image. In the study by Wawrzyk-Bochenek et al., GLCM was effectively used for quantitative analysis of discolouration reduction after microneedle mesotherapy using vitamin C. The same authors [30] confirmed the effectiveness of hyperpigmentation therapy using kojic acid by monitoring changes in GLCM contrast and homogeneity values. After the treatments, a reduction in GLCM contrast was observed in about 83% of cases and an increase in skin homogeneity in about 67% of cases.

In the present study, the GLCM contrast showed a stronger correlation with the CEA score than homogeneity, which suggests its greater usefulness in the quantitative assessment of erythema. The statistically significant correlation between the subjective CEA scores and objective GLCM parameters confirms the consistency and mutual complementarity of these skin condition assessment methods. High correlation values (R = 0.81–0.94 for contrast and negative for homogeneity) show that an increase in the intensity of erythema evaluated visually is associated with the increase in irregularity of the skin texture visible in imaging analysis. The achieved correspondence between the results of subjective and objective methods emphasises the importance of using advanced tools for analysing dermatological images to support traditional clinical scaling, thus increasing the repeatability and precision of measurements.

### Limitations

Attention should be paid to the limitations of this study. First, there was no control group (e.g., apparent treatment or untreated area), which reduces the certainty that all observed changes were caused only by IPL. Second, this study had a small research group consisting of 20 volunteers; in the future, we plan further research on a larger group. Third, although the images were coded, the potential lack of complete blinding in the analysis cannot be completely excluded. Fourth, the predominance of female participants and the small male subgroup restrict the ability to draw firm conclusions about gender-specific effects.

## 5. Conclusions

It was observed that three polychromatic light treatments provided very good clinical effects in reducing erythema, which is one of the symptoms of rosacea. The effects persisted at a high level for at least three months. Both the CEA scale as a subjective method commonly used by dermatologists and GLCM analysis as a method of objective analysis of the skin texture fully confirm the effectiveness of the therapy. The integration of these two types of diagnostic tools—a traditional clinical assessment and advanced imaging analysis—can be a path for future research and clinical practice in the assessment of skin erythema.

## Figures and Tables

**Table 1:** Parameters of individual treatments used for each volunteer.

Volunteer Number	Energy Density J/cm^2^		
	Treatment 1	Treatment 2	Treatment 3
1	12	14	17
2	14	16	17
3	14	15	16
4	13	15	17
5	13	14	16
6	13	15	17
7	14	16	17
8	12	14	16
9	13	15	16
10	12	14	16
11	12	14	16
12	12	13	15
13	13	15	16
14	12	14	16
15	14	15	16
16	13	15	16
17	12	14	15
18	12	14	15
19	12	14	16
20	12	14	16

**Table 2:** Correlation of the mean CEA score and GLCM contrast and homogeneity calculated for the entire face before the treatments (T0), four weeks after three treatments (T1) and three months after three treatments (T2) (Spearman).

	N	R	t(N − 2)	*p*
CEA T0 and T0 Contrast	20	0.943	12.057	<0.001
CEA T1 and T1 Contrast	20	0.839	6.547	<0.001
CEA T2 and T2 Contrast	20	0.814	5.947	<0.001
	N	R	t(N − 2)	*p*
CEA T0 and T0 Homogeneity	20	−0.828	−6.256	<0.001
CEA T1 and T1 Homogeneity	20	−0.769	−5.097	<0.001
CEA T2 and T2 Homogeneity	20	−0.788	−5.433	<0.001

## Data Availability

Dataset available on request from the authors.
